# Selective activation of microglia in spinal cord but not higher cortical regions following nerve injury in adult mouse

**DOI:** 10.1186/1744-8069-4-15

**Published:** 2008-04-18

**Authors:** Fuxing Zhang, Kujumon I Vadakkan, Susan S Kim, Long-Jun Wu, Yuze Shang, Min Zhuo

**Affiliations:** 1 Department of Physiology, Faculty of Medicine, University of Toronto, University of Toronto Centre for Study of Pain, 1 King's College Circle, Toronto, Ontario M5S 1A8, Canada

## Abstract

Neuronal plasticity along the pathway for sensory transmission including the spinal cord and cortex plays an important role in chronic pain, including inflammatory and neuropathic pain. While recent studies indicate that microglia in the spinal cord are involved in neuropathic pain, a systematic study has not been performed in other regions of the central nervous system (CNS). In the present study, we used heterozygous *Cx3cr1*^*GFP*/+^mice to characterize the morphological phenotypes of microglia following common peroneal nerve (CPN) ligation. We found that microglia showed a uniform distribution throughout the CNS, and peripheral nerve injury selectively activated microglia in the spinal cord dorsal horn and related ventral horn. In contrast, microglia was not activated in supraspinal regions of the CNS, including the anterior cingulate cortex (ACC), prefrontal cortex (PFC), primary and secondary somatosensory cortex (S1 and S2), insular cortex (IC), amygdala, hippocampus, periaqueductal gray (PAG) and rostral ventromedial medulla (RVM). Our results provide strong evidence that nerve injury primarily activates microglia in the spinal cord of adult mice, and pain-related cortical plasticity is likely mediated by neurons.

## Introduction

Microglia are the resident macrophages in the CNS. They exert important functions such as phagocytosis of cellular debris and/or neuronal signal processing when activated, through communications with neurons, immune cells and glial cells [1-3]. Activation of microglia occurs in most pathological processes. The activation is accompanied by changes in morphology, upregulation of immune surface antigens, and production of cytotoxic or neurotrophic molecues [1,4,5]. It has been found that spinal microglia was activated after peripheral nerve injury [6,7], and the activated microglia might release many bioactive molecules such as cytochines, chemikines and neurotrophic factors (like brain-derived neurotrophic factor (BDNF)), which then could modulate the excitability of spinal neurons [7-9].

Recent evidence clearly indicates that nerve injury-induced plasticity is not just limited in the DRG and spinal dorsal horn neurons, and inhibition of these signalling proteins at lower level (DRG and spinal dorsal horn) is not sufficient to prevent or inhibit neuropathic pain [10-15]. The anterior cingulate cortex (ACC), a critical region for pain perception, undergoes long-term plastic changes after peripheral inflammation or nerve injury [10,14,16,17]. Consistently, clinic studies of patients with neuropathic pain showed significant changes or heightened activities in the ACC [15,18]. Consistent with neuronal changes, activation or increased expression of immediate early genes in the ACC neurons, such as c-fos, Egr1 and 3',5'-cyclic adenosine-monophosphate response element-binding protein (CREB) have been reported after different injury conditions (inflammation, nerve injury or amputation) [10,12,14]. In addition to changes in the supraspinal structures, there is increasing evidence suggesting that endogenous pain modulatory systems including descending facilitatory system also undergo long-term plastic changes after injury [14,15,19-21].

In contrast to the large extent of neuronal changes observed in CNS, less is known about whether changes in brain microglia happen under physiological or pathological conditions. Recent studies on acute brain slice *in vitro *or in brain *in vivo *showed that resting microglia move their processes toward the source of exogenously applied ATP or tissue injury [22-26], but it is unresponsive to glutamate, GABA application or activity-dependent long-term potentiation (LTP) [27]. These findings indicate that microglial cells in the brain may not respond to neuronal plasticity triggered by peripheral injury [15]. In order to determine whether nerve injury induces microglial cell changes along the pain-processing pathway including cortical areas and pain-modulatory descending pathways, we performed a systematic study on microglial morphology in these pain-related structures from the spinal cord to brain, using transgenic mice in which all microglia are labelled by green fluorescence protein (GFP) after replacing the first 390 bp of *Cx3cr1 *gene with a cDNA encoding enhanced GFP [28]. Heterozygous *Cx3cr1*^GFP/+ ^mice were used, since the fractalkine receptor function is intact with GFP expression [29]. We examined microglia in the CNS in transgenic mice receiving control or nerve ligation.

## Methods

### Animals

Eight heterozygous *Cx3cr1*^*GFP*/+ ^ten-week old mice were used [28]. These mice were derived from BALB/c *Cx3cr1*^*GFP*/*GFP *^intercrossed with C57BL/6. All animals were housed on a 12 h/12 h light/dark cycle with food and water provided *ad libitum*. The experimental protocols were approved by The Animal Care and Use Committee at the University of Toronto.

### Surgical procedure

Mice were divided into two groups, control (sham surgery) and common peroneal nerve (CPN) ligated. The surgical procedure was performed as previously described [30]. Briefly, animals were anaesthetized by intraperitoneal injection of 10 μl per gram body weight of a mixture of 0.5 mL xylazine (20 mg/mL, Bayer, Toronto, Canada) and 1.3 mL ketamine (100 mg/mL. Bimeda MTC, Cambridge, Ontario) in 8.2 mL of saline. 1 cm skin incision was made in the left hind leg to expose the CPN. The CPN was ligated with chromic gut suture (5-0, Ethicon, Somerville, New Jersey) without disturbing or occluding the blood vessel. The skin was sutured using 5-0 silk suture and cleaned with povione iodine. Sham surgery was conducted in the same manner but the nerve was not ligated. All animals were kept in a 37°C warming chamber connected to a pump (Gaymar T/Pump, Orchard Park, NY) for at least 1 h post surgery.

### Measurement of mechanical allodynia

Allodynia was tested under non-restrained conditions. Mice were allowed to acclimatize to the transparent cylindrical container for 30 min before testing. A threshold stimulus was determined by an animal's hind paw withdrawal upon application of a von Frey filament (Stoelting, Wood Dale, IL) to the point of bending over the dorsum of the hind paw. Mechanical sensitivity of the animal to the innocuous pressure of a 0.4 mN von Frey Filament (No. 2.44) was scored and repeated every 5 min for up to 10 times. Positive responses included prolonged hind paw withdrawal and licking or biting of the hind paw. Mechanical allodynia was tested on 1, 3 and 7 days post surgery.

### Histology

Seven days after surgery, the animals were anesthetized and perfused with 0.1 mol/L phosphate buffered saline (PBS, pH 7.2–7.4; 0.9% NaCl) followed by 4% paraformaldehyde in 0.1 mol/L phosphate buffer via the ascending aorta. The mice were then immediately decapitated. The brain, L2 to L5 lumbar spinal segments and the associated DRGs on both sides were removed. All these structures were cryoprotected with PBS containing 30% sucrose at 4°C overnight. Coronal brain sections were serially cut in cryostat. Every fourth section 50 or 25 μm thick were collected for observation under the confocal laser scanning microscope (Olympus, BX61WI, Japan) and epifluorescence microscope (Olympus BX51, Japan), respectively. The sections were then mounted onto gelatin coated slides, and were air-dried and coverslipped. Following fluorescent examination, Nissl staining was performed to reveal the structures of the brain or spinal cord.

### Quantification and Data analysis

Microglia quantification was performed on sections of 25 μm thickness. The number of microglia within confines of each anatomically demarcated nucleus was counted with the help of computer interfaced digital image analysis system. This was performed by an observer who was blind to the animal treatment. Only the cell bodies were taken into account. Images for microglia density evaluation were captured using epifluorescence microscope under 4× objective through software *Image-Pro Plus *(5.0., Media cybernetics, Silver Spring, USA). Comparable brain sections containing the nucleus of interest between mice were used, and counting was made on three sections per mouse. When counting, image contrast was adjusted such that the background level just disappeared, and the same cutoff level was used for all images. Cell type identification and counting were performed with epifluorescence microscopy under 20× objective. Three randomly sampled areas containing at least 50 cells in a structure were examined for each of the three sections per mouse. The percentage of a particular cell type was obtained by calculating the ratio of the cell number of this type to the total number of microglial cells examined. Six sections of L4 spinal segment per mouse were chosen for spinal microglia count. Spinal microglia counting was similar to that of brain microglia, except that all cells from each lamina were examined. For DRG neuron area calculation, only those with clear nucleus and neuronal profile were considered, and any cell with an area of less than 100 μm^2^, which may probably indicate glial cell, were not taken into account.

All data are presented as Mean ± SEM (Table 1, 2) per section. Statistical difference was tested between two sides in a group using student's t-test or between different structures through one-way ANOVA.

**Table 1:** Quantitative analysis of microglia density and percentage of particular microglia type in pain-related brain structures (Ipsi. and Contral. indicate surgery and intact sides, respectively).

				**Cell type (%)**							
				
**Region**		**Density (number/mm**^2^**)**		**Ramified**		**Hypertrophied**		**Mono-polar**		**Bipolar**	
		**Ipsi.**	**Contral.**	**Ipsi.**	**Contral.**	**Ipsi.**	**Contral.**	**Ipsi.**	**Contral.**	**Ipsi.**	**Contral.**
**ACC**	control	191.0 ± 27.6	200.0 ± 38.4	95.7 ± 0.3	94.4 ± 0.5	0	0	4.3 ± 0.3	4.4 ± 0.3	0	1.2 ± 0.4
	injury	181.7 ± 13.6	188.7 ± 13.5	92.1 ± 0.4	92.3 ± 1.7	0	0	5.7 ± 0.3	4.4 ± 0.2	2.2 ± 0.7	3.3 ± 1.7
**Prefrontal**	control	193.3 ± 27.6	199.7 ± 28.9	94.6 ± 0.9	95.6 ± 0.9	0	0	4.1 ± 0.4	4.3 ± 0.9	1.3 ± 0.2	0
**Cortex**	injury	184.7 ± 14.5	180.3 ± 18.9	89.9 ± 2.5	90.1 ± 2.2	2.6 ± 1.5	0	6.3 ± 0.3	7.3 ± 1.3	1.2 ± 0.7	2.6 ± 1.4
**Somatosensory**	control	169.3 ± 17.6	152.0 ± 17.6	97.2 ± 0.9	98.8 ± 0.5	0	0	2.1 ± 0.5	1.2 ± 0.5	0.7 ± 0.4	0
**Cortex**	injury	184.0 ± 24.5	178.3 ± 13.6	92.8 ± 2.4	94.9 ± 0.6	0	0	4.5 ± 2.1	5.1 ± 0.6	2.7 ± 0.6	0
**Insular cortex**	control	189.0 ± 11.8	209.2 ± 16.8	94.5 ± 0.6	96.6 ± 0.6	0	0	4.8 ± 0.3	2.9 ± 0.3	0.7 ± 0.3	0.5 ± 0.5
	injury	176.7 ± 22.6	174.3 ± 18.2	98.0 ± 0.3	96.3 ± 0.6	0	0	1.5 ± 0.3	2.7 ± 0.3	0.5 ± 0.3	1.0 ± 0.6
**Amygdala**	control	194.0 ± 21.7	176.7 ± 26.3	94.2 ± 1.2	98.0 ± 1.3	1.1 ± 0.6	0	2.6 ± 0.2	2.0 ± 1.3	2.1 ± 0.6	0
	injury	184.3 ± 12.5	183.3 ± 14.2	93.9 ± 1.8	96.3 ± 2.0	0	0	3.3 ± 0.9	3.7 ± 2.0	2.7 ± 0.9	0
**Hippocampus**	control	182.3 ± 20.8	183.3 ± 13.6	93.3 ± 0.7	92.5 ± 0.9	0	0	4.3 ± 0.7	4.3 ± 0.5	2.4 ± 0.4	3.2 ± 0.5
	injury	176.3 ± 12.7	185.0 ± 15.0	91.1 ± 2.3	88.7 ± 2.2	0	1.5 ± 0.6	7.0 ± 1.2	6.7 ± 0.6	1.9 ± 1.2	3.1 ± 3.0
**Thalamus**	control	145.4 ± 34.1	124.2 ± 37.1	96.6 ± 0.6	94.0 ± 0.9	0	0	3.4 ± 0.6	4.5 ± 0.9	0	1.5 ± 0.2
	injury	131.3 ± 46.5	139.4 ± 37.9	86.7 ± 2.3	89.8 ± 2.1	0	0	6.6 ± 1.2	7.3 ± 1.2	6.7 ± 1.2	2.9 ± 0.9
**PAG**	control	105.7 ± 12.3	104.0 ± 20.4	92.7 ± 2.6	90.4 ± 3.1	0	0	4.0 ± 1.6	7.6 ± 1.6	3.3 ± 1.1	1.9 ± 0.8
	injury	130.0 ± 25.0	125.3 ± 15.5	88.4 ± 1.8	86.1 ± 2.3	0	0	7.3 ± 1.2	7.5 ± 1.4	4.3 ± 0.8	6.4 ± 1.0
**RVM**	control	79.3 ± 12.5	77.7 ± 17.0	91.8 ± 0.9	90.5 ± 1.2	0	0	5.1 ± 0.3	4.3 ± 0.3	3.1 ± 0.6	5.2 ± 0.9
	injury	78.7 ± 14.6	82.3 ± 12.3	89.5 ± 2.6	91.3 ± 0.7	0	0	6.4 ± 1.4	4.0 ± 0.9	4.1 ± 1.2	4.7 ± 0.7

**Table 2:** Quantitative analysis of microglia density and percentage of particular microglia type in spinal cord (Ipsi. and Contral. indicate surgery and intact sides, respectively).

				**Cell type (%)**							
				
**Lamina**		**Density (number/mm**^2^**)**		**Ramified**		**Hypertrophied**		**Mono-polar**		**Bipolar**	
		**Ipsi.**	**Contral.**	**Ipsi.**	**Contral.**	**Ipsi.**	**Contral.**	**Ipsi.**	**Contral.**	**Ipsi.**	**Contral.**
**I**	control	373.3 ± 14.2	327.0 ± 47.8	91.7 ± 1.7	90.0 ± 0.9	0	0	8.3 ± 1.7	10.0 ± 0.9	0	0
	injury	692.0 ± 22.5	373.3 ± 16.2 (**)	18.7 ± 2.7	89.4 ± 2.9 (**)	37.3 ± 3.8	0 (**)	14.8 ± 1.5	10.6 ± 2.9	29.2 ± 5.6	0
**II**	control	275 ± 39.6	228.7 ± 27.6	84.4 ± 2.8	83.3 ± 1.9	0	0	7.1 ± 2.1	8.3 ± 1.6	8.4 ± 2.1	8.3 ± 1.6
	injury	532.3 ± 15.1	251 ± 57.8 (**)	18.2 ± 1.5	86.0 ± 2.3 (**)	52.7 ± 2.2	0 (**)	21.8 ± 1.3	6.0 ± 3.4	7.3 ± 0.3	8.0 ± 1.5
**III**	control	203.0 ± 14.7	214 ± 30.1	85.0 ± 2.3	90.5 ± 1.4	0	0	8.8 ± 2.6	4.8 ± 0.3	6.2 ± 0.3	4.7 ± 2.1
	injury	376.3 ± 60.2	253.0 ± 46.3 (**)	47.8 ± 2.0	88.9 ± 1.2 (**)	26.1 ± 0.9	0 (**)	21.7 ± 4.7	5.3 ± 1.8	4.3 ± 2.9	5.8 ± 0.6
**IV**	control	182.2 ± 59.8	194.0 ± 45.7	85.7 ± 1.6	87.7 ± 1.7	0	0	9.5 ± 0.8	8.3 ± 1.7	4.8 ± 2.0	4.0 ± 0.9
	injury	235.7 ± 18.0	203.0 ± 39.5	81.3 ± 1.2	85.7 ± 1.4	3.1 ± 1.2	0	6.3 ± 0.9	7.1 ± 1.2	9.3 ± 3.2	7.2 ± 0.3
**V**	control	184.3 ± 12.7	191.3 ± 60.1	89.5 ± 1.5	86.4 ± 2.3	0	0	5.3 ± 1.2	8.1 ± 1.8	5.2 ± 0.3	5.5 ± 0.6
	injury	237.3 ± 30.1	190.3 ± 24.8	78.4 ± 1.7	81.5 ± 1.6	2.7 ± 0.9	0	10.8 ± 1.4	7.4 ± 0.8	8.1 ± 2.3	11.1 ± 2.0
**VI**	control	147.0 ± 13.5	137.3 ± 17.6	85.7 ± 1.7	86.7 ± 3.8	0	0	7.1 ± 0.8	6.7 ± 1.5	7.1 ± 1.2	6.6 ± 2.4
	injury	162.7 ± 3.5	146.0 ± 20.1	76.0 ± 1.8	80.0 ± 2.1	0	0	13.3 ± 1.5	9.8 ± 2.4	10.7 ± 1.9	10.2 ± 1.2
**VII**	control	101.0 ± 12.5	116.7 ± 15.5	81.6 ± 2.3	85.7 ± 1.6	0	1.6 ± 1.6	10.2 ± 1.8	7.9 ± 1.4	8.1 ± 0.6	4.8 ± 0.7
	injury	171.7 ± 9.7	118.7 ± 14.1 (**)	75.7 ± 2.0	84.0 ± 2.3	1.3 ± 1.3	0	11.7 ± 1.4	10.0 ± 1.9	11.3 ± 1.2	6.0 ± 2.7
**VIII**	control	124.3 ± 46.2	114.0 ± 34.4	85.7 ± 2.3	84.2 ± 1.8	0	0	9.5 ± 0.9	5.3 ± 0.5	4.8 ± 1.5	10.5 ± 2.0
	injury	134.3 ± 10.4	149.0 ± 23.5	80.0 ± 1.2	83.7 ± 2.2	0	0	6.7 ± 0.7	5.5 ± 1.0	13.3 ± 1.2	10.8 ± 1.4
**IX**	control	145.3 ± 14.1	156.3 ± 19.2	87.0 ± 2.9	87.3 ± 1.5	0	0	4.3 ± 0.6	8.5 ± 1.2	8.7 ± 2.3	4.2 ± 0.5
	injury	535.0 ± 61.9	138.6 ± 12.1 (**)	8.9 ± 2.7	86.7 ± 2.1 (**)	75.0 ± 2.8	3.7 ± 0.7(**)	12.5 ± 1.5	4.8 ± 1.2	3.6 ± 1.2	4.8 ± 0.5
**X**	control	175.3 ± 42.3	167.0 ± 49.4	77.8 ± 1.2	82.5 ± 1.4	0	0	11.1 ± 0.9	12.5 ± 0.9	11.1± 2.0	5.0 ± 1.2
	injury	263.0 ± 40.6	165.3 ± 49.7	76.7 ± 2.9	75.0 ± 0.3	0	0	15.0 ± 1.5	12.5 ± 1.2	8.3 ± 2.3	12.5 ± 0.9

## Results

### Neuropathic pain behaviour following nerve injury

**Figure 1:**
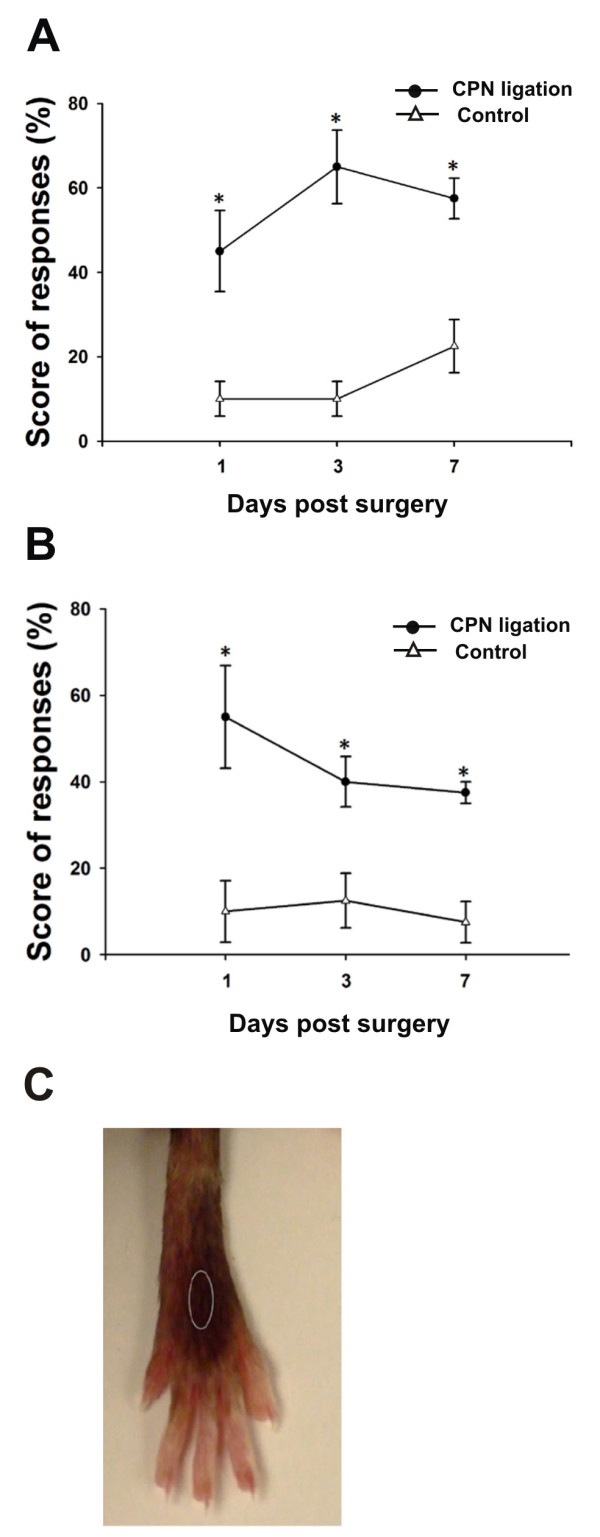
**Tactile allodynia post unilateral common peroneal nerve ligation**. (A, B) Ipsilateral (A) and contralateral (B) hind paw withdrawals in response to tactile stimulus were plotted against the time. Significant higher scores of allodynia were observed on the nerve injured side than the intact side. (C) Circled area indicates the location receiving von Frey filament touch. * *P *< 0.05 (n = 4).

In the present study, we used a mouse model of neuropathic pain established in our lab recently [30]. Compared with other animal models mimicking peripheral neuropathic pain, CPN ligation model is technically easy, causes less muscle injury and muscle weakness, and will not hinder normal motor function. As previously reported, we observed a significant mechanical allodynia in mice receiving the CPN ligation. From post-operative day 1 onwards, the frequency of hind paw withdrawals elicited by mechanical stimulus was always significantly higher in CPN ligated animals than in control animals (*P *< 0.05, n = 4 for each group) (Fig. 1). After behavioural allodynia test, we then examined microglia along the somatosensory pathway from the DRG to pain-related brain areas in control mice and mice receiving the CPN ligation.

### Characterization of different types of microglial cell

**Figure 2:**
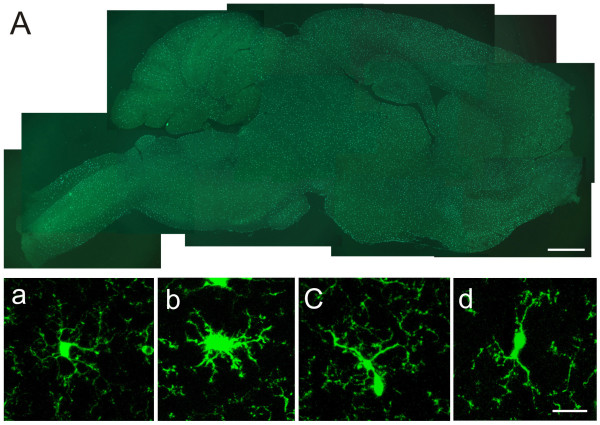
**Photomicrographs showing microglia in the brain**. (A) Montage of a saggittal section showing the distribution of brain microglia in control mice. (a-d) Different types of brain microglial cells observed under confocal laser scanning microscopy. Brain microglial cells were classified as ramified (a), hypertrophied (b), mono-polarized (c) and bipolarized (d). Hypertrophied microglia were defined as having large soma, and short, thick and radially projecting processes. Ramified microglial cells were defined as possessing thin, slender, radially projecting processes with well-developed ramifications. Monopolarized microglial cells were defined as having one thick process with well developed ramifications extending toward one direction. Bipolarized microglial cells were defined as having two thick processes emanating from the opposing poles of the cell and projecting in the opposite directions. Bar = 1 mm in A, and 20 μm in a-d.

**Figure 3:**
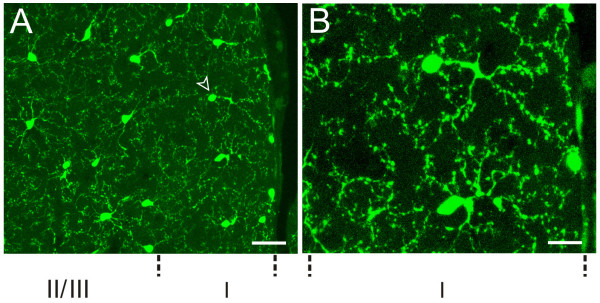
**Microglia in anterior cingulate cortex (ACC)**. (A) Microglial cells were evenly distributed in ACC of control mice, the centrifugally projecting ramified processes of neighbor microglial cells didn't overlap. The arrowhead indicates the monopolarized cell. (B) High power image of the microglial cell indicated by arrowhead in A. Bar = 30 μm in A and 12 μm in B.

In general, a significant number of microglial cells, labeled by GFP, were observed throughout the whole brain region of control mice under both epifluorescence and confocal laser scanning microscopes (see Fig. 2A). It has been reported that the morphological phenotypes vary among microglia population, especially under pathological conditions [31]. In control mice, we found that the GFP labeling in individual microglia differed in the intensity among subcellular compartments. The cell body, however, is always the most intensely labeled. Individual microglial cells are different in shape, each cell displaying a unique morphology. Here we grouped them into four different types of microglial cells on the basis of their morphology and projecting direction of the processes: ramified, amoeboid, unipolar and bipolar microglial cells. The ramified microglial cells are defined as the ones possessing slender, radially projecting processes with similar thickness, length and ramification (Fig. 2a). The amoeboid (or hypertrophied) microglia are characterized by large soma, short/thick and radially projecting processes with few ramifications (see Fig. 2b). The third type of microglia cells is the polarized microglia. They were characterized by several ramified processes, but only one or two of which are much better developed. This group of cells can be subdivided into two subgroups: unipolar and bipolar microglia (Fig. 2c,d). The former has elongated or pyriform soma, with the predominant process displaying well-developed arborizations and showing directional extension (Fig. 2c, 3). Other processes, if any, are dwarfed and poorly developed. Bipolar microglia had spindle-like cell bodies and the soma had just two processes which project from the opposing poles of the cell body (Fig. 2d). In control mice, more than 90% of the microglia are ramified cells, and less than 10% belong to polarized cells. There are a few hypertrophied microglia found in the control mice (see Table 1).

### Microglia in pain-related brain areas of control mice

**Figure 4:**
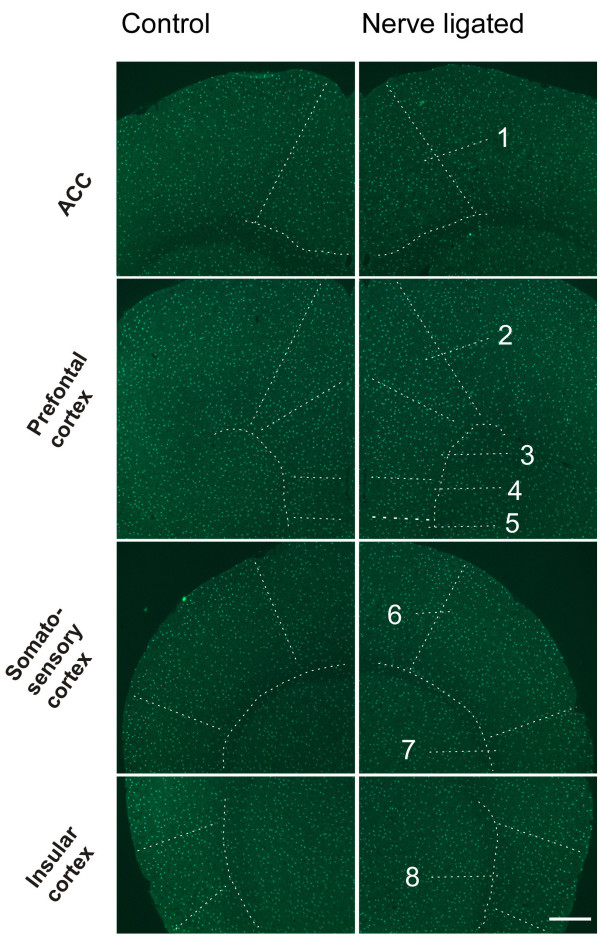
**Microglia in pain-related cortices of control mice**. Left column, sham-operated; right column, CPN ligated. The structures are indicated by arrow or enclosed by dashed lines. 1, ACC (Anterior cingulate cortex); 2, Cingulate cortex, area 1; 3, Prelimbic cortex; 4, Infralimbic cortex; 5, Dorsal peduncular cortex; 6, S1 (primary somatosensory cortex); 7, S2 (secondary somatosensory cortex); 8, Insular cortex. Bar = 400 μm.

**Figure 5:**
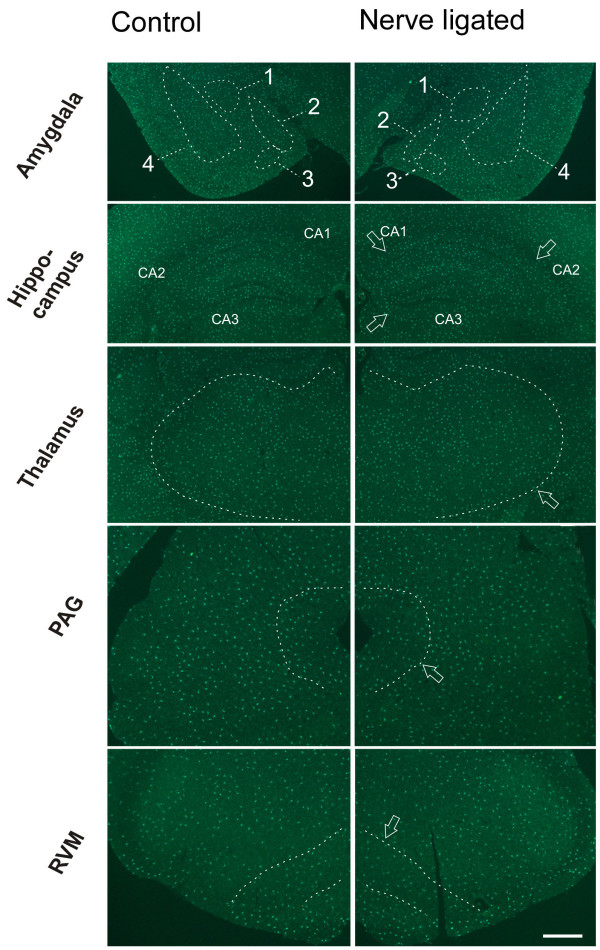
**Microglia in amygdala, hippocampus, thalamus, PAG and RVM of control mice**. Left column, sham-operated; right column, CPN ligated. The structures are indicated by arrow or enclosed by dashed lines. 1, Central amygdaloid nucleus; 2, medial amygdaloid nucleus; 3, Posteromedial amydaloid nucleus; 4, Lateral amygdaloid nucleus, basolateral and basomedial amygdaloid nuclei. Bar = 800 μm.

Pain activates many regions of the CNS. Here we decided to focus on the following five major cortical areas that are reported to be important in chronic pain: the ACC, prefrontal cortex (PFC), somatosensory cortex (S1 and S2) and insular cortex (IC) [12,16,18,19]. Two major regions that are known to contribute to pain-related spatial memory/emotion (hippocampus) and fear/anxiety (amygdala) were also examined [11]. In addition, the endogenous pain modulatory systems including the periaqueductal gray (PAG) and rostroventral medial medullary (RVM) were examined [32]. In order to understand the general morphological properties of microglia in these structures, we first performed systematic characterization of microglia in the control mice. As shown in Figures 3, 4, 5, we found a grid-like distribution of microglial cells in the pain-related brain structures. Each microglial cell and its branched processes occupied a micro-territory, and the processes from adjacent cells didn't overlap. Microglial cells formed a regularly spaced network (Fig. 3). Although the GFP-labelled microglia seemed to be homogeneously distributed throughout the brain, they did show some difference in the microglia density among the aforementioned brain structures (sham-operated side, *F*_(8,26) _= 3.705, *P *= 0.01; contralateral side, *F*_(8,26) _= 3.389, *P *< 0.05) (Table 1). The density of microglia ranged from estimated 80 cells per mm^2 ^in the RVM (the lowest one among examined areas) to the 200 cells per mm^2 ^in the ACC (Table 1, Fig. 4, 5). The distribution of microglia on the sham-operated and contralateral side of brain were not significantly different (*P *> 0.05, n = 3) (Table 1).

For cortical areas, we found microglial cells are similarly distributed in pain-related cortical areas, including the ACC, PFC, S1/S2, and IC, with an average density of 191.0 ± 27.6, 193.3 ± 27.6, 169.3 ± 17.6 and 189.0 ± 11.8 cells per mm^2^, respectively, for sham-operated side; and 200.0 ± 38.4, 199.7 ± 28.9, 152.0 ± 17.6 and 209.2 ± 16.8 cells per mm^2^, respectively, for the contralateral side (Table 1, Fig. 4). We found that microglial cells are similarly distributed in both parts of the brains, and no significant difference was detected between two hemispheres (*P *> 0.05, n = 3) (Table 1, Fig. 4). Among them, around 95% microglial cells are ramified, less than 5% are cells of other types. There are no significant difference in density among these brains cortices (sham-operated side, *F*_(3,11) _= 0.247, *P *= 0.861; contralateral side, *F*_(3,11) _= 0.922, *P *= 0.473) (Table 1). In the ACC, microglia cells are distributed in each layer, and no obvious difference can be observed visually between two sides (Fig. 3, 4).

For the hippocampus and amygdala, we found that microglial cells had a similar density as cortical areas (sham-operated side, *F*_(5,17) _= 0.185, *P *= 0.963; contralateral side, *F*_(5,17) _= 0.688, *P *= 0.642) (Table 1, Fig. 5). The average density for hippocampus and amygdala was 182.3 ± 20.8 and 194.0 ± 21.7 cells per mm^2^, respectively, on sham-operated side; and 183.3 ± 14.2 and 176.7 ± 26.3 cells per mm^2^, respectively on the contralateral side. No significant difference was observed between two sides of either structures (*P *> 0.05, n = 3) (Table 1). Again, the majority of cells are ramified microglia, with an average percentage of over 93%. Less than 7% of the microglia were of other cell type.

In the thalamus, which is involved in both sensory and pain signal transmission, the microglia densities ipsilateral and contralateral to the sham-operated side were 145.4 ± 34.1 and 124.2 ± 37.1 cells per mm^2^, respectively, and there was no significant difference between them (*P *> 0.05, n = 3) (Fig. 5). Over 94% of the microglial cells were ramified cells and less than 6% were hypertrophied or polarized.

In the PAG and RVM, an average density of microglia of 105.7 ± 12.3, and 79.3 ± 12.5, respectively, on the sham-operated side; and 104.0 ± 20.4 and 77.7 ± 17.0, respectively, on the contralateral side were observed (Fig. 5). Compared with microglia in forebrain structures, the microglia density in PAG showed no significant difference (sham-operated side, *F*_(7,23) _= 1.855, *P *= 0.145; contralateral side, *F*_(7,23) _= 2.114, *P *= 0.102). However, microglial density in the RVM showed significant difference when compared to the forebrain structures (sham-operated side, *F*_(7,23) _= 2.961, *P *= 0.034; contralateral side, *F*_(7,23) _= 3.004, *P *= 0.032) (Table 1). Similar to other brain regions examined, no significant difference in density between two sides was detected (*P *> 0.05, n = 3) (Table 1). More than 91% of the microglia in these structures were ramified cells and less than 9% were of other cell type.

### Microglia in brains of nerve injured mice

To see whether microglial phenotypes changed after CPN ligation, we also examined microglia in the pain-related brain areas of CPN ligated mice. Similar to the cases of control mice, many microglial cells were observed following CPN ligation in the ACC, PFC, S1, S2, IC, amygdala, hippocampus, thalamus, PAG, and RVM (Table 1). The mean density in these pain-related brain areas were observed to range from around 80 per mm^2 ^in RVM to 190 per mm^2 ^in ACC (Table 1). To test whether the CPN ligation could induce microglial change, we compared the four values in each structure representing microglial density of different sides of the two animal groups (Table 1), one-way ANOVA test showed that CPN ligation did not induce any significant change of microglia density in each of these pain-related brain structures. In CPN ligated mice, four types of microglial cells were also observed. More than 85% of the microglial cells in the pain-related brain areas were ramified (Table 1). Other types of cells accounted for less than 15%. When compared with control group, the percentage of each type of microglial cells in the individual structures showed no significant difference.

### Microglia in the Spinal cord

**Figure 6:**
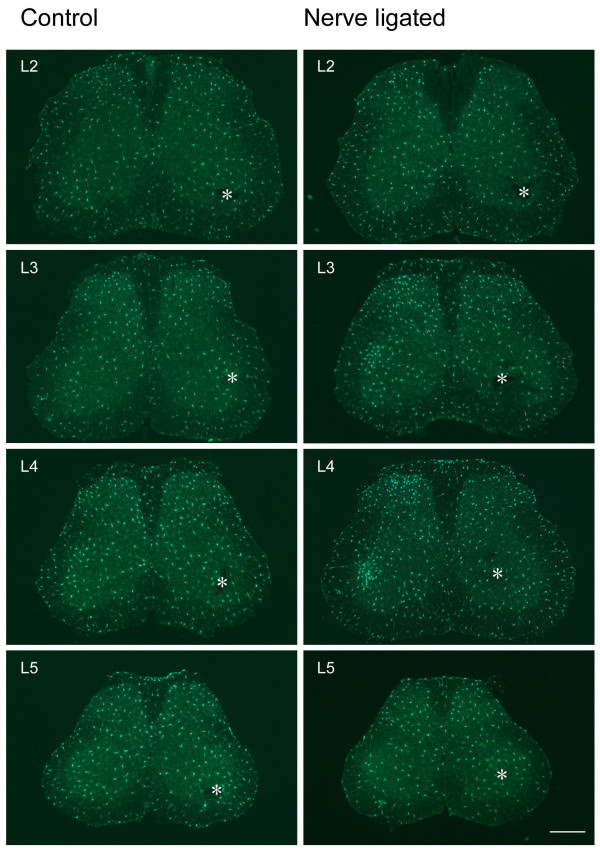
**Microglia is activated in spinal segments of nerve injured mice but not control mice**. Left column, sham-operated; right column, CPN ligated. Nerve injury evoked an ipsilateral increase of microglial cells in superficial layers of L2 to L4 spinal dorsal horns, and also in deep layers of L3 and L4 spinal segments. No visible change occurred in L5 spinal segment. Asterisks indicate intact side. Bar = 350 um.

It has been reported that spinal microglia could be activated following different nerve injury paradigms [3,6,7]. Most of previous studies were carried out in adult rats. To investigate the spinal microglia change following CPN ligation in adult mice, we examined the density and morphological phenotypes in spinal cord of control and CPN ligated mice. From L2 to L4, no visually difference was demonstrated between two sides of spinal cord in control mice (Fig. 6 left column, Table 2).

**Figure 7:**
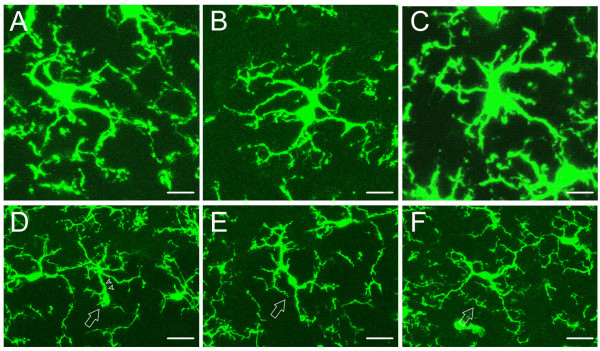
**Different morphological phenotypes of spinal microglial cells observed with confocal laser scanning microscopy**. Four types of spinal microglial cells were detected in control mice. (A, B) Ramified microglial cells have radially projecting processes which are long, thin with fine ramifications. (C) Hypertrophied cell has large soma and thick, short and radially projecting processes with fewer ramifications. (D) Mono-polarized microglia (arrow) has one main process (arrowhead) which is thick and projects toward one direction. (E, F) Bipolarized microglia (arrow) usually has spindle-like cell body and two main processes emanating from the opposing poles of the cell body and projecting in opposite directions. Bar = 10 μm in A-C, 20 μm in D-F.

In spinal cord of control mice, the same four types of microglial cells as those observed in brain were identified (Fig. 7), that is, ramified (Fig. 7A,B), hypertrophied (Fig. 7C), monopolarized (Fig. 7D) and bipolarized (Fig. 7E,F). Over 80% of the microglia were ramified with slender, fine processes, lower than 20% were polarized, and nearly no hypertrophied cells were encountered. These values showed no significant difference between two sides of spinal cord (*P *> 0.05, n = 3, *t*-test) (Table 2).

**Figure 8:**
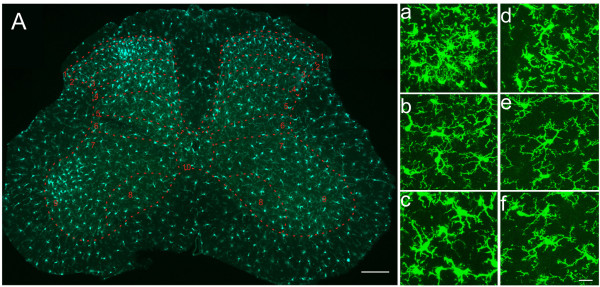
**Activation of microglia in L4 spinal cord segment following nerve injury**. (A) Epifluorescence imgage of L4 spinal cord segment following nerve injury. Left half side in the image represents the nerve injured side. Laminae of dorsal and ventral horns are indicated by the numbers. Note the difference of microglia between two sides. (a-f) Confocal laser scanning microscopy observation of microglia from the same laminae as shown by panel A. (a-c) Microglial cells from lamina II, IV and IX of nerve injured side, respectively. (d-f) Microglial cells from lamina II, IV and IX of the intact side, respectively. Bar = 400 μm in A, 20 μm in a – f.

Following CPN ligation, an obvious increase of microglia on the injured side was visualized from L2 to L4 spinal segments, while L5 showed no visual difference (Fig. 6 right column). The increased microglia were mainly distributed in the medial part of dorsal horn (Fig. 8A). An aggregate of microglia were obvious in dorsal horn, including lamina I and outer part of lamina II. In addition, activation of microglia was also clearly seen in ventral horn and lamina IX (Fig. 6, right column, L3 and L4; Fig. 8). In L4 which manifested the most dramatic increase of microglia, mean density increase of microglia was observed on the injured side in laminae I to V, and laminae VII, IX and X (Table 2, Fig. 8). Student's *t*-test showed that microglia density in laminae I, II, III, VII and IX on the injured side were significantly increased than that on intact side (*P *< 0.05, n = 3) (Table 2, Fig. 8A).

In laminae I to III and lamina IX of CPN ligated mice, the percentages of hypertrophied and ramified microglial cells were significantly increased on the injured side when compared to the contralateral side (*P *< 0.05, n = 3, *t*-test), while other types of cells showed no significant change between two sides for most laminae (Table 2).

### GFP-labelled glia in DRG

**Figure 9:**
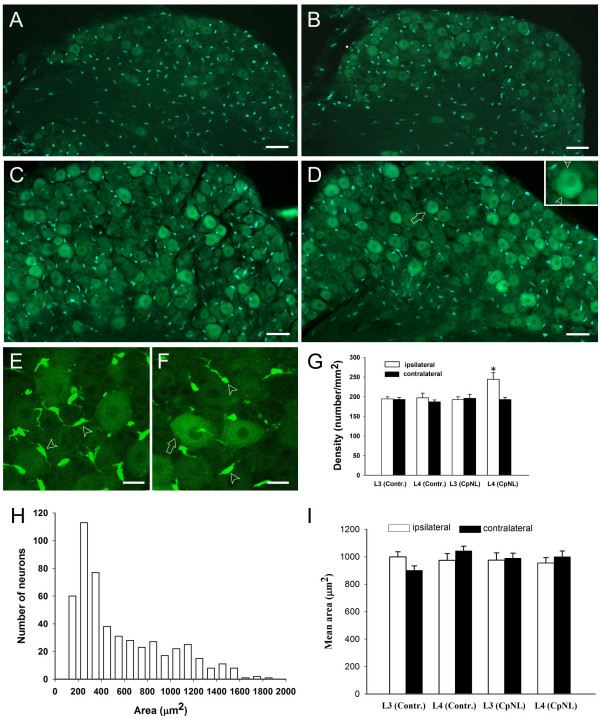
**DRG manifest both GFP-labeled neurons and glia**. (A-B) GFP-labelling in L4 DRGs of control mice on sham sugery and intact sides, respectively. (C-D) GFP-labelling in L4 DRGs of nerve injured mice on injured and contralateral sides, respectively. Higher density of glia cells in injured DRG (C) contrasts that of intact DRG (D). The GFP-labeled neuron in (D) indicated by arrow is shown in the inset at a higher magnification, arrowheads point to the satellite cells in close apposition to the neuron. (E-F) Confocal laser scanning microscopic images of injured and intact L4 DRGs, respectively. Arrowheads and arrow point to the Schwann cells and satellite cell, respectively. (G) The density of glial cells was significantly higher in injured L4 DRG than in its contralateral counterpart (p < 0.05, n = 3). "Contr." and "CpNL" indicate control and nerve injured mice, respectively. (H) Number of different-sized L4 DRG neurons in intact side of control mice (bin size = 100). (I) the size of L3/L4 DRG neurons with strong labeling showed no change after nerve injury (p > 0.05, n = 3). The area is expressed as Mean ± SEM (μm^2^/per neuron). "Contr." and "CpNL" indicate control and nerve injured mice, respectively. Bar = 75 μm in A-B; 50 μm in C-D, 20 μm in E-F.

To explore whether nerve injury could induce DRG glial cell changes, we examined GFP-labeled DRG glia cells in both groups of mice. GFP-labeled glia cells were observed in DRGs of control mice, as shown in L2 to L5 DRGs of both sides (Fig. 9A,B). Obviously, DRG glial cells differed in shape from spinal cord or brain microglia, they are tadpole-like with mean area of 49.3 ± 19.6 μm^2 ^in control mice (n = 50 cells from intact L4 DRG). While most glial cells were scattered among DRG neurons, emanating one or just few processes, some GFP-labeled glial cells were closely apposed to the DRG neurons (Fig. 9).

Following nerve ligation, individual DRG glial cells showed neither morphological nor distributional change (Fig. 9C,D and 9G). The number of glial cells in DRG of the injured side was increased (Fig. 9C,D and 9G). Semi-quantitative analysis revealed that glial cell density in injured L4 DRG is significantly higher than its intact counterpart (Fig. 9G), while L3 DRGs showed no difference in density between two sides (Fig. 9G).

It is surprisingly to find that many DRG neurons, from large-, medium- to small-sized ones, were also observed to be GFP-positive (Fig. 9A–F). To our knowledge, this is the first evidence suggesting the expression of fractalkine receptors in the DRG cells. Interestingly, the DRG neurons with strongest GFP labeling were almost exclusively those with mean area around or over 900 μm^2 ^(Fig. 9A–F). In order to know the morphological properties of these neurons in the context of whole DRG, we measured the size of 509 DRG neurons, including both labeled and unlabelled, from intact L4 segments of control mice, and the total number of different sized neurons was plotted against the area. We found that the most strongly GFP-labeled DRG neurons exhibited larger size (Fig. 9H). The size of these neurons showed no statistical difference between two sides in either control or CPN ligated mice (Fig. 9I). In addition, the density of these neurons was 257.6 ± 38.2 on the injured side and 250.6 ± 20.1 cells per mm^2 ^on the intact side. These values were not significantly different (*P *> 0.05, n = 3, *t*-test) between two sides in CPN ligated mice.

## Discussion

In the present study, we have systemically examined the distribution and morphology of microglial cells in the pain-related neural pathways in both control mice and mice with nerve injury. Similar to previous reports in rats, we have shown that peripheral nerve injury caused activation of microglial cells in the spinal cord dorsal horn. In addition, we also observed a clear activation microglial cells in the ventral horn of the spinal cord, a possible network link to the dorsal horn activity at the same side of the spinal cord. The employment of *Cx3cr1*^*GFP*/+ ^mice allows direct observation of microglia with epifluorescence microscopy, without immunolabelling for microglia. This reduced the possibility of missing some microglial cells, as is the case when immunostaining for microglia specific antigens, such as cytokines, CD4, ED1, MHCII or OX-42 are used for microglia identification [31,33]. Our results provide the first systematic characterization of microglia in the CNS, and we show that the activation of microglia is unlikely driven by neuronal activity. Many supraspinal structures that are known to play critical roles in chronic pain failed to show any sign of activation of microglial cells after nerve injury, suggesting that the contribution of microglia to chronic neuropathic pain may be limited to the spinal cord dorsal horn.

Recent studies in the ACC found that peripheral injury including nerve injury and inflammation induced long-term plastic changes in sensory synaptic transmission [15]. In the present study, we didn't observe significant change of microglia density in pain-related brain areas following nerve ligation (Table 1). The findings is consistent with our recent study that microglia was not activated by neuronal activities as well as LTP in brain slices [27]. However, this did not necessarily mean that microglia in these areas remain inactive under neuropathic pain conditions. Alteration at the subcellular and biochemical levels might exist. The biochemical changes of brain microglia are supported by a recent study that showed several microglial markers such as OX-42, TLR4 and CD14 were upregulated following intraplantar injection of complete Freunds adjuvant (CFA) [34].

Microgliosis occurs in spinal cord following nerve injury. The present study demonstrated an increased density of microglia in spinal cord on nerve injured side, this agrees with previous findings by using different nerve injury paradigms [3,6,7]. Following peroneal nerve ligation, dorsal horn laminae I to III manifested higher density of microglia. Anatomically, these areas are where primary sensory afferents innervating mechanoreceptors and nociceptors project. It was reported that nerve injury rendered primary afferents more excitable and discharging spontaneously [35]. Therefore, the excitatory signals from the injured nerve to these spinal areas may be one of the primary factors triggering microglia activation. In addition, biochemical changes along the neuronal sensory pathway may be an additional cause for microglia activation. We found a cluster of microglia occurred in the motor nucleus region of L3 or L4 spinal ventral horn. It has been reported that cranial nerve transaction induced microgliosis in the associated brain motor nucleus, playing a possible role of phagocytosis [7]. For instance, increased number of microglia gathered in hypoglossal nucleus and facial nucleus following axotomy of the corresponding nerves [36]. Future studies are clearly needed to identify the factors that contribute to the activation of microglia.

Following common peroneal nerve ligation, L4 DRG on the injured side showed an increase of glial cells. Similar findings were also found after transaction or chronic constriction of sciatic nerve. DRG gliosis was reported to be possibly originated from invasion of macrophages which are recruited to perform phagocytosis [37]. In contrast with L4 DRG, injured L3 DRGs did not show glial change, but L3 spinal microglia did. This mirrored the fiber components comprising this nerve and may agreed with spinal sites that primary afferents of this nerve project to. It is well known in human that common peroneal nerve is comprised of the spinal segments from L4 across down to second sacral spinal segment. Taking this together with our findings, we speculated that CPN ligation in mice induced no L3 DRG injury, but L3 and its upper spinal segments received abnormal signals from lower injured DRGs.

Monocytes, some NK cells and microglial cells expressed the receptor, CX(3)CR1, and all these cells were reported to be labeled by GFP in *Cx3cr1*^*GFP*/+ ^mice [28,29,38,39]. Based on this, we speculate that the GFP-labelled DRG neurons, as seen in the present study, also expressed CX(3)CR1. It has been reported that neuropathic pain induced great biochemical change in the medium- to large-sized DRG neurons, including calcitonin gene-related peptide (CGRP), TRPV1 and alpha 2-adrenoreceptor up-regulation [40,41]. In the present study, we didn't find morphological change in the larger DRG neurons exhibiting the brightest GFP labelling. The reason may be that 7 days post nerve ligation is not long enough for DRG neurons to develop visible change. In agreement with this, DRG neurons showed no degeneration or cell death until 2 weeks after sciatic nerve transection [42]. Future studies are clearly needed to investigate the physiological roles of the fractalkine receptors in the DRG cells.

In summary, we have performed systemic mapping of microglia in major pain-related brain areas in control mice and mice with nerve injury. In addition to confirming the activation of spinal microglia cells after nerve injury, we did not find any other activation of microglial cells in supraspinal structures. Our results provide strong evidence that nerve injury caused a rather regional selective activation of microglia in the CNS, and suggest that activation of microglia cells are not likely due to abnormal neuronal activity triggered by nerve injury.

## Authors' contributions

FZ carried out the surgery and histology studies, perform statistical analysis and drafting the manuscript. KIV carried out the part of histology work. SSK performed the surgery and behavior test. LJW participated in the design of the study and drafting the manuscript. YS performed the quantification and partial histology work. MZ conceived of the study, and participated in its design and coordination and drafting the manuscipt. All authors read and approved the final manuscript.
